# Effects of Rhein on Bile Acid Homeostasis in Rats

**DOI:** 10.1155/2020/8827955

**Published:** 2020-11-09

**Authors:** Zhong Xian, Jingzhuo Tian, Lianmei Wang, Yushi Zhang, Jiayin Han, Nuo Deng, Suyan Liu, Yong Zhao, Chunyin Li, Yan Yi, Dunfang Wang, Jing Meng, Chen Pan, Aihua Liang

**Affiliations:** Key Laboratory of Beijing for Identification and Safety Evaluation of Chinese Medicine, Institute of Chinese Materia Medica, China Academy of Chinese Medical Sciences, Beijing 100700, China

## Abstract

Rhein, the active ingredient of rhubarb, a medicinal and edible plant, is widely used in clinical practice. However, the effects of repeated intake of rhein on liver function and bile acid metabolism are rarely reported. In this work, we investigated the alterations of 14 bile acids and hepatic transporters after rats were administered with rhein for 5 weeks. There was no obvious injury to the liver and kidney, and there were no significant changes in biochemical indicators. However, 1,000 mg/kg rhein increased the liver total bile acid (TBA) levels, especially taurine-conjugated bile acids (t-CBAs), inhibited the expression of farnesoid X receptor (FXR), small heterodimer partner (SHP), and bile salt export pump (BSEP) mRNA, and upregulated the expression of (cholesterol 7*α*-hydroxylase) CYP7A1 mRNA. Rhein close to the clinical dose (10 mg/kg and 30 mg/kg) reduced the amounts of TBAs, especially unconjugated bile acids (UCBAs), and elevated the expression of FXR and multidrug resistance-associated protein 3 (Mrp3) mRNA. These results denote that rhein is relatively safe to use at a reasonable dose and timing. 30 mg/kg rhein may promote bile acid transport and reduce bile acid accumulation by upregulating the expression of FXR mRNA and Mrp3 mRNA, potentially resulting in the decrease in serum UBCAs.

## 1. Introduction

Bile acids play an important role in regulating the metabolism balance of lipids *in vivo* [1]. They are converted from cholesterol in liver by a series of enzymes, and they maintain a dynamic balance through the uptake and efflux of hepatocellular transporter as well as the enterohepatic circulation [2]. Some liver diseases and drug-induced liver injury are often accompanied by obstacles in the synthesis, metabolism, and excretion of bile acids, potentially leading to the accumulation of bile acids. However, excessive amounts of bile acid results in hepatocyte injury, even liver cirrhosis and necrosis [3]. The homeostasis of bile acid levels is closely related to the occurrence and development of liver diseases. Despite the limitations of clinical chemistry in sensitivity and specificity at the early stage of liver diseases, there are significant alterations of bile acids in plasma and urine [4]. Thus, bile acids can be used as potential biomarkers for liver injury and dysfunction [5].

Rhein (PubChem CID: 10168) is widely found in medicinal plants such as rhubarb, *Sennae folium*, *Semen cassiae*, and *Polygonum multiflorum*. The amount of rhein in these plants are approximately 4.7 mg/g (rhubarb) [6], 0.29 mg/g (*Polygonum multiflorum*) [7], 0.24 mg/g (*Semen cassiae*) [8], and 0.4 mg/g (*Sennae folium*) [9], suggesting that the amount of rhein in rhubarb is relatively high. Recent studies have indicated that rhein has lipid-lowering, anti-inflammatory, antitumor, and antihepatic fibrosis effects as wells as reduces blood glucose and improves renal interstitial fibrosis [10–12]. These medical plants are broadly used all over the world. In China, approximately 10% (800) of more than 8,000 proprietary Chinese medicines contain rhubarb [13]. In the list of health care products published by the State Food and Drug Administration of China in 2016, 66 kinds contain rhubarb, 253 kinds contained aloe, 440 species contain *Semen casssiae*, and 282 species contain *Polygonum multiflorum*. Most of them are used to reduce blood lipids and lose weight [14]. In addition to being used as a laxative in Europe, rhubarb is frequently used in food as a vegetable or in the production of desserts, jams, and fruits [15]. Moreover, the cultivated area of rhubarb in some Nordic countries is approximately 60 hectares. Meanwhile, the rhubarb cultivation area in the United States and Canada is approximately 7 times the cultivated area of Europe [16]. Rhubarb is widely popular in North America as an ingredient in pie [17]. Rhubarb has the effects of promoting dampness and relieving jaundice [18], anti-inflammatory activity, and kidney protection, preventing and treating high blood lipids and cholestasis, ameliorating fibrosis and hepatic encephalopathy, and promoting blood circulation and hemostasis [19]. Anthraquinones are the active constituents of rhubarb, and they include rhein, emodin, chrysophanol, emodin methyl ether, and aloe-emodin [20]; meanwhile, studies have indicated that rhein showed a higher bioavailability and was more easily absorbed and exposed than other anthraquinones [21]. The pharmacokinetic study of rhein showed that the peak plasma concentration of rhein was 2.45 *μ*g/mL within 25 min after oral administration of a dosage of 11.9 mg/kg of rhein in rats, and its elimination half-life was 144 min [22]. However, the effects of repeated intake of rhein on liver function and bile acid metabolism are rarely reported. Therefore, the present study was designed to investigate the influences of different doses of rhein on liver and bile acid metabolism and provide information for the reasonable use of rhein.

## 2. Materials and Methods

### 2.1. Chemicals and Reagents

**Figure 1:**
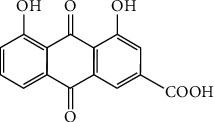
Chemical structure of rhein.

Rhein (Figure 1) was purchased from Beijing Saibaicao Technology Co., Ltd., with purity of >98%. All reference bile acids purchased were of high purity. TCA (taurocholic acid) was purchased from Cayman Chemical Company (Ann Arbor, Michigan, US). T-*α*-MCA, TDCA, GUDCA, GHDCA, and *β*-MCA were purchased from Toronto Research Chemicals (Toronto, Canada). GCDCA, GDCA, and GCA were purchased from Nanjing Shenglide Technology Co., Ltd. (Nanjing, China). THDCA, CA, UDCA, HDCA, CDCA, and DCA were purchased from the National Institutes for Food and Drug Control (NIFDC, Beijing, China). Deuterated internal standards DCA-d4, GDCA-d4, and TDCA-d4 were obtained from Cambridge Isotope Laboratories (Tewksbury, US).

### 2.2. Animals and Experimental Procedure

Twenty male Sprague-Dawley rats at 6-8 weeks were obtained from Vital River Laboratory Animal Technology Co., Ltd. (Beijing, China). They were maintained in a controlled environment. The animals were randomly divided into 0, 10, 30, and 1,000 mg/kg rhein groups. Rhein was suspended in 0.5% sodium carboxymethyl cellulose solution, and the volume of administration was 10 mL/kg. Rats were dosed by a gastric gavage once a day for 5 weeks, while the rats in the control group were administered an equal volume of sodium carboxymethyl cellulose solution. Rats were anesthetized with sodium phenobarbital by intraperitoneal injection. Blood samples were collected from the abdominal aorta by syringe under the condition of fasting 16 h, and then, serums and livers were obtained. The serum samples were used for the analysis of bile acids and biochemical indexes. The livers were used for pathological examination, the analysis of gene expression, and the detection of bile acids. This study was approved by the committee on the ethics of animal experiments of the Institute of Chinese Materia Medica, China Academy of Chinese Medical Sciences (Permit Number: 20152018).

### 2.3. Analysis of Bile Acids in Serum and Liver by Liquid Chromatography-Mass Spectrometry (LC/MS)

#### 2.3.1. Conditions of Chromatography and Mass Spectrometry

The mobile phase consisted of 0.01% formic acid in water (A) and 0.01% formic acid in acetonitrile (B). The gradient of A was as follows: 75–60% A (0–9 min), 60–35% A (9–13 min), 35–1% A (13–16 min), and 1-75% A (16–18 min). The mass spectrometer was operated in negative mode with the MRM (multiple-reaction monitoring) function for the quantification [23], and more details on the MRM conditions are shown in Supplementary Table S1. The mass spectrometer was operated with the source and desolvation temperatures set at 120°C and 350°C, respectively. The curtain gas was 40 psi; the ion spray voltage was -4500 V; the probe temperature was 600°C; nitrogen was used as collision gas and set at medium; ion source gas 1 and ion source gas 2 were all 40 psi.

#### 2.3.2. Sample Preparation for LC/MS Analysis

For the serum samples, Oasis-HLB SPE (solid-phase extraction) columns were used for sample extraction. Deuterated standards of CA, GCA, and TCA were used as internal standards (ISs). First, 100 *μ*L serum samples were spiked with 10 *μ*L of the working solution of ISs (1 *μ*g/mL) and added 890 *μ*L water containing 0.01% formic acid. After vortexing for 2 min, the samples were loaded onto SPE columns preactivated with 1 mL methanol, followed by 2 mL 0.01% formic acid water. Then, the loaded cartridges were washed with 1 mL 10% MeOH, 1 mL 0.05% formic acid, and 1 mL 45% methanol and eluted with 3 mL MeOH. For each 100 mg of liver, 600 *μ*L of cold methanol and 10 *μ*L of the 1 *μ*g/mL working solution of ISs were added. Then, the liver tissues were homogenized twice for 30 s in MP FastPrep-24 sample preparation system. Tubes were centrifuged at 6,000 rpm/min for 10 min at 4°C, and the supernatants were transferred to clean tubes. A second BA extraction was performed using 400 *μ*L of cold methanol. Finally, the two extraction supernatants were combined and centrifuged at 15,600 rpm/min for 10 min at 4°C. Then, the supernatants were evaporated to dryness by nitrogen (N_2_) at 37°C and reconstituted in 100 *μ*L methanol, and 1 *μ*L was injected into the AB SCIEX QTRAP 6500 LC/MS system.

#### 2.3.3. Calibration and Quality Control Standard Preparation

The calibration standards ranging from 2 to 10,000 ng/mL and QC (quality control) standards (100, 1,000, and 10,000 ng/mL) were then disposed of according to the sample preparation process depicted above. The linear regression parameters which were obtained for serum and liver bile acids are shown in Supplementary Table S2 and Table S3, respectively. Five replicates of each QC point were analyzed to evaluate the intra- and interday accuracy and precision (Supplementary Table S4 and Table S5). This process was repeated 3 times over 3 days to evaluate the interday accuracy and precision. The extraction recoveries of 14 bile acids are shown in Supplementary Table S4.

#### 2.3.4. Individual Bile Acid Analysis

The individual bile acid (BA) analysis was performed by LC/MS. Chromatographic separation of the bile acid was performed on an ACQUITY UPLC (ultraperformance liquid chromatography) BEH column (2.1 × 100mm, 1.7 *μ*m) (Waters Corp., Milford, US) maintained at 35°C at a flow rate of 500 *μ*L/min. The deuterated standards d4-TCA, d4-GCA, and d4-CA were used as ISs for TCA, GCA, and CA, respectively. Peak integration and quantification were performed using Analyst 1.6.2 software. Data were processed with SIMCA-P software, version 12.0.

### 2.4. Biochemical Assay and Histopathology

AST, ALT, ALP, *γ*-GT, CHO, TBIL, BUN, and CRE were examined using a TBA-40FR automatic biochemistry analyzer (Toshiba, Japan). Histopathological examination of the liver was performed in all rats. Organ samples were fixed with neutral buffered formalin, embedded in paraffin, sectioned, and stained with HE. The histomorphology was examined under a light microscope (Olympus, Japan).

### 2.5. Quantitative Real-Time PCR Analysis

Total hepatic RNA was prepared by a total RNA kit (OMEGA, Georgia, USA) in accordance with instructions. An aliquot of 1 *μ*g RNA was applied for reverse transcription with oligo-dT primer (TOYOBO, OSAKA, Japan). Quantitative real-time PCR was performed using the Roche 480 instrument (Roche, Mannheim, Germany) and SYBR Green PCR Master Mix (Roche, Mannheim, Germany) for the subsequent genes with the corresponding primers (Sangon Biotech, Beijing, China) (Supplementary Table S6). Quantification was conducted using the ΔΔCT method [24]. The quantity of messenger RNA was normalized with the internal standard GAPDH.

### 2.6. Statistical Analysis

The results are shown as the mean ± SD. Data were analyzed using SPSS 16.0 software, and differences between control group and administration groups were analyzed by one-way analysis of variance (ANOVA). Bile acid data were also determined by OPLS-DA (orthogonal partial least-squares discrimination analysis) using SIMCA-P 12.0 software. The data of the rhein treatment groups were compared with the control. Values significantly different from the control are indicated as ^∗∗^*p* < 0.01 and ^∗^*p* < 0.05.

## 3. Results

### 3.1. Physical Effects of Rhein

**Figure 2:**
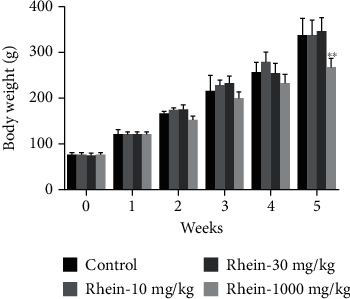
Body weight after rats treated with rhein for 5 weeks. ^∗^*p* < 0.01, ^∗∗^*p* < 0.05, compared with the control group.

The body weights of rats in the 1,000 mg/kg group were notably reduced compared with the control group (*p* < 0.01, Figure 2). In addition, the urine volume of rats in the 10 mg/kg and 30 mg/kg groups was remarkably increased compared with the control (data not shown). The relative liver weights were not different from control. No diarrhea was found during the study period, and there were no other abnormal signs in the rats during the administration period.

### 3.2. Multivariate Regression Analysis of Bile Acids in Serum and Livers

**Figure 3:**
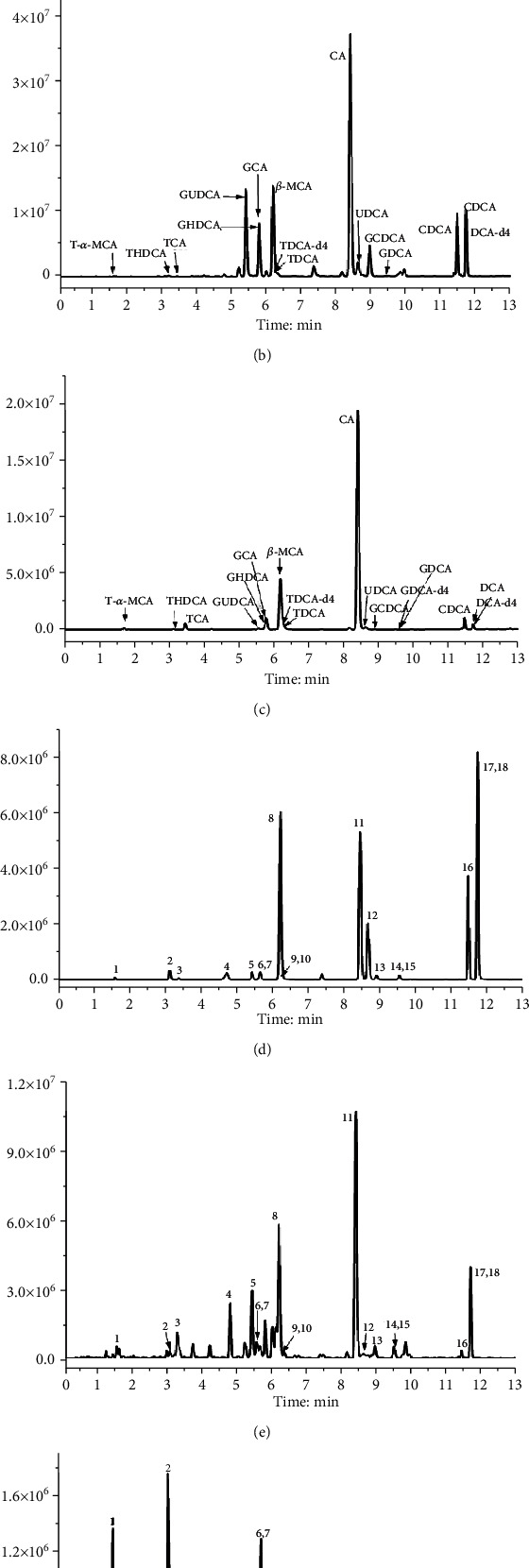
UPLC-MS/MS chromatogram of bile acids in serum and livers. Bile acid standards in serum samples (a), serum samples in the control group (b), and serum samples in the 1,000 mg/kg group (c) and bile acid standards in the hepatic samples (d), hepatic samples in the control group (e), and hepatic samples in the 1,000 mg/kg group (f).

**Figure 4:**
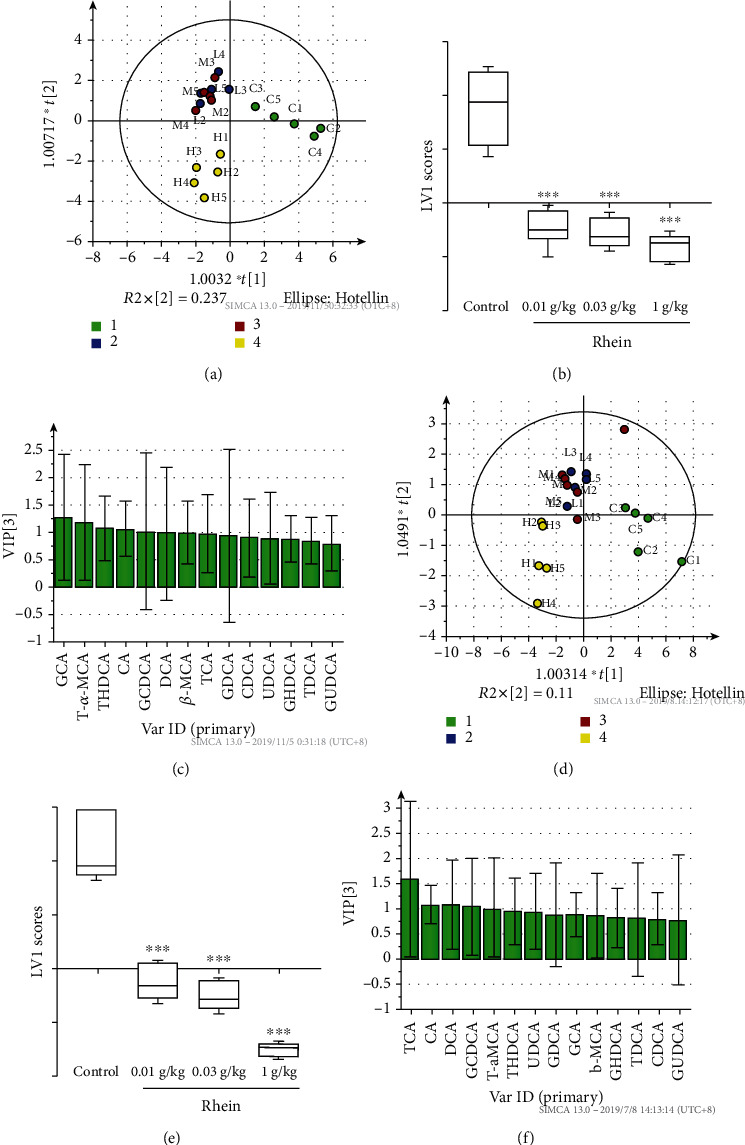
Multivariate data analysis of bile acid profiles in the serum and liver. The OPLS-DA score plots demonstrated complete separation of the samples between groups in the serum (a) and liver (d). The green points represented the control, while the blue, red, and yellow points represented the 10 mg/kg, 30 mg/kg, and 1,000 mg/kg rhein group, respectively, as shown on the plots. The t1 scores in the serum (b) and liver (e) are shown, respectively, according to the OPLS-DA score plots. The VIP plots of OPLS-DA highlighted the discriminatory species in the serum (c) and liver (f).

The LC/MS chromatograms of 14 serum and liver bile acids are shown in Figure 3. The specific concentrations of serum and liver bile acids are shown in Table 1. OPLS-DA (orthogonal partial least-squares discrimination analysis) revealed a good segregation between the treatment groups and the control (Figures 4(a) and 4(d)). Although the 10 and 30 mg/kg groups were not completely separated, they were still divided from the control. The 1,000 mg/kg rhein group was not only better distinguished from the control but also separated from the 10 mg/kg and 30 mg/kg groups (Figure 4). These results indicate that rhein markedly altered the composition and levels of serum and liver bile acids. The analysis of the animal latent variable 1 (LV1) scores for both serum (Figure 4(b)) and liver (Figure 4(e)) showed that the bile acid levels in the rhein groups differed from the control. The VIP (variable importance in projection) value was used to assess potential markers (Figures 4(c) and 4(f)), and a VIP value above 1.0 implied that the independent variable had a more important role in the interpretation of the dependent variable [25]. Using this criterion, we screened TCA, THDCA, T-*α*-MCA, *β*-MCA, and GCA in serum and TCA, T-*α*-MCA, CA, DCA, and GCDCA in liver as potential markers.

### 3.3. Serum Biochemical Parameters Analysis

The serum biochemical indexes (Table 2) were investigated. The results show that there were no marked differences in serum ALT, AST and CHO levels. The level of serum ALP in the 30 mg/kg group was lower than that in the control, but the change was not dose-dependent. The treatment with 1,000 mg/kg rhein reduced the serum TBIL and *γ*-GT levels (*p* < 0.05). However, the reduction of the above indicators did not have toxicological significance. The results imply that rhein has no significant toxicological effect.

### 3.4. Histopathological Examination of Liver

**Figure 5:**
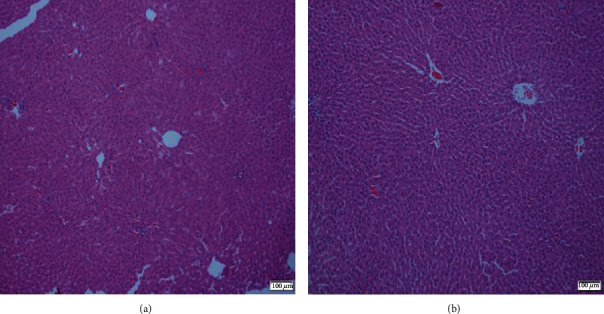
Histomorphological changes in the livers of rats with or without rhein treatment. Paraffin-embedded liver sections were stained with hematoxylin and eosin (HE). (a) Control group and (b) 1,000 mg/kg rhein group. The magnification is 100x.

Histopathological examination of the liver showed inflammatory infiltration and fat droplets at 1,000 mg/kg rhein. Furthermore, mild ductular proliferation was found in 1 of the 5 rats after treated with 1,000 mg/kg rhein (Figure 5(b)). However, histological abnormalities were not observed in the control, 10 mg/kg, and 30 mg/kg groups.

### 3.5. Serum Bile Acids Profiles after Treatment with Rhein

**Figure 6:**
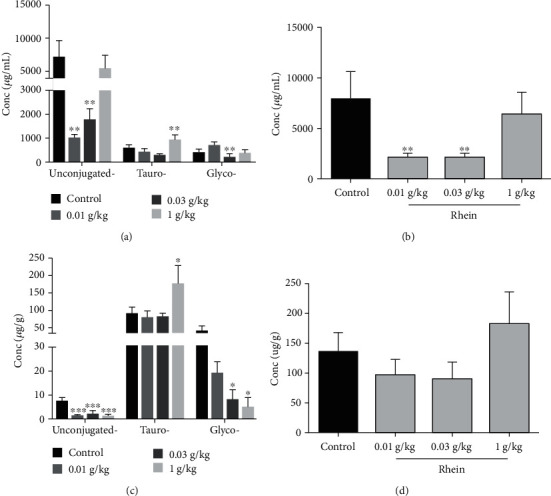
Alterations in the composition of bile acids in the serum and liver after treated rats with rhein. Serum concentrations of t-CBAs, g-CBAs, and UCBAs (a) and concentrations of TBAs (b) in different groups. In addition, concentrations of t-CBAs, g-CBAs, and UCBAs (c) and concentrations of TBAs (d) in the liver of different groups. Data are presented as mean ± SD of 5 rats. ^∗^*p* < 0.05, ^∗∗^*p* < 0.01, compared with the control group.

The amounts of 14 bile acids in serum were determined (Table 1), and the contents of g-CBAs, t-CBAs, UCBAs, and TBAs (sum of the 14 bile acids) were classified and analyzed (Figures 6(a) and 6(b)). The results showed that the level of UCBAs in serum was the highest. The composition ratio and contents of serum bile acids in rats were significantly affected by rhein. And g-CBAs (especially GCDCA, GDCA, and GCA, *p* < 0.05 or *p* < 0.01) as well as t-CBAs (especially T-*α*-MCA and THDCA, *p* < 0.05 or *p* < 0.01) were decreased. The dose of 1,000 mg/kg rhein significantly reduced the amounts of 3 UBCAs (DCA, CDCA, *β*-MCA, *p* < 0.05 or *p* < 0.01) while slightly elevating the CA level. Meanwhile, the high dose of rhein affected the g-CBA and t-CBA contents remarkably. Two g-CBAs (GUDCA 375%↓, GCDCA 149.5%↓, and GDCA 509.2%↓) and two t-CBAs and GHDCA (170.7%↑) and two t-CBAs (T-*α*-MCA 115.5%↑, TCA 108.7%↑) were decreased and increased, respectively. The results demonstrate that different doses of rhein displayed a diverse influence on bile acid homeostasis, and 10 mg/kg and 30 mg/kg rhein may be beneficial to its therapeutic effect of reducing bile acids.

### 3.6. Hepatic Bile Acid Profiles after Treatment with Rhein

The LC/MS chromatograms of 14 liver bile acids are shown in Figures 3(d)–3(f). From Figures 6(c) and 6(d), we can find that the t-CBAs accounted for the majority of TBA, while the proportion of UCBAs was the lowest. The hepatic TBAs were slightly decreased; meanwhile, the TBA concentrations were elevated when the dose of rhein was increased to 1,000 mg/kg. Rhein notably decreased the contents of UDCA, DCA, *β*-MCA, CA, and CDCA in liver (Table 1). The contents of 5 g-CBAs were reduced, showing a decreased trend with the elevation of the dose of rhein. For the t-CBAs, 10 mg/kg and 30 mg/kg rhein remarkably reduced the TDCA level and elevated the T-*α*-MCA level (*p* < 0.01). The dose of 30 mg/kg rhein could also significantly reduce the level of THDCA versus the control. When the dose of rhein was up to 1,000 mg/kg, it not only had a more notable effect in decreasing the TDCA and THDCA levels and the increasing T-*α*-MCA level but also significantly elevated the content of TCA. These results suggest that large doses of rhein can increase TBA levels in the liver, mainly t-CBAs.

### 3.7. Impact of Rhein on Hepatic Bile Acid Transport and Gene Expression

**Figure 7:**
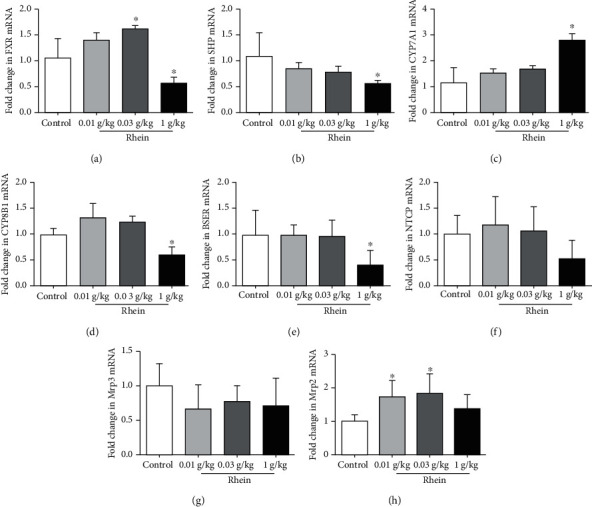
Expressions of genes involved in hepatic bile acids regulation. Quantitative real-time PCR analysis was performed to measure the expressions of genes in livers, including FXR (a), SHP (b), CYP7A1 (c), CYP8B1 (d), BSEP (e), NTCP (f), Mrp2 (g), and Mrp3 (h). Data are presented as mean ± SD of 5 rats. ^∗^*p* < 0.05, compared with the control group.

To investigate the mechanism of abnormal alterations of bile acids induced by rhein, we analyzed the gene expression of liver bile acid receptor FXR and the transporters associated with bile acid synthesis, transport, and excretion by quantitative real-time PCR. It can be seen from Figure 7 that treatment with 10 mg/kg rhein upregulated the expression of FXR mRNA by 38% and remarkably elevated the expression of Mrp3. 30 mg/kg rhein had a remarkable effect on the expression of FXR and Mrp3 mRNA. However, the expression of SHP, CYP7A1, CYP8B1, NTCP (Na^+^-dependent taurocholic cotransporting polypeptide), BSEP, and Mrp2 (multidrug resistance-associated protein 2) mRNA was not significantly affected by these two doses. When rhein was increased to 1,000 mg/kg, the expression of FXR and SHP mRNA were repressed, and there was a significant difference compared with the control (*p* < 0.05). At the same time, the expression of CYP7A1 was upregulated (*p* < 0.05). But 1,000 mg/kg rhein could significantly suppress the expression of BSEP mRNA (*p* < 0.05). However, there was no significant effect on NTCP, Mrp2, and Mrp3 mRNA expression. These results indicate that the effects of different doses of rhein lead to diverse effects on the gene expression involved in bile acid homeostasis.

## 4. Discussion

According to previous reports, rhein exerts significant anti-inflammatory effects, antioxidant effects, lipid-lowering effects, osteoarticular protection, and antidiabetic activities and improves renal interstitial fibrosis [10]. The maximum daily dose of rhubarb is 15 g according to Chinese Pharmacopoeia [18]; meanwhile, the content of rhein in rhubarb is 0.47%-2.11% [6, 26]. Therefore, the amount of rhein that an adult may ingest per day when taking 15 g rhubarb is ranging from 70.5 mg to 316 mg, which is equivalent to 7.4 mg/kg to 33 mg/kg in rats according to the dose conversion method [27]. Considering the fluctuation of the amounts of rhein in medicinal materials, we chose 10 mg/kg rhein, close to the clinical equivalent dose, as the lowest dose in this study, and designated another 30 mg/kg and 1,000 mg/kg rhein (approximately equivalent to 3 times and 100 times of the clinical dose, respectively) to explore the possible influence of different doses of rhein on bile acid homeostasis. In this study, treatment of rats with rhein for 5 weeks did not result in obvious injury to the liver and kidney, except for mild liver histological changes caused by 1000 mg/kg rhein, and no toxicological effects were found in the blood biochemical indicators, implying that rhein has low toxicity. The concentrations of 14 bile acids were obtained by UPLC-MS/MS and found that rhein notably altered the constitution and amounts of serum and liver bile acids.

To elucidate the mechanism of the changes of bile acid induced by rhein, the gene expression associated with bile acid homeostasis was analyzed by quantitative real-time PCR. Bile acid synthesis takes place in the liver via two different pathways. The classical pathway is catalyzed by CYP7A1, the rate-limiting enzyme [28] deciding the contents of bile acid synthesis and regulated by the FXR [29], to produce most bile acids. Meanwhile, FXR is an important regulator; it not only regulates bile acid synthesis but also plays an important role in bile acid transport and excretion [30, 31]. FXR induces SHP leading to inhibition of CYP8B1 and CYP7A1, which ultimately reduces the contents of bile acids. In addition, FXR also regulates CYP7A1-mediated bile acid synthesis by directly regulating the expression of the fibrous growth factor FGF15 (mouse)/FGF19 (human) [32, 33]. In this study, the expression of SHP mRNA was not consistent with that of FXR mRNA in low and medium dose of rhein. It may be that FXR regulates CYP7A1-mediated bile acid synthesis by regulating the expression of FGF15, but it still needs to be verified. Bile acids are secreted from the liver into the bile canaliculus via the BSEP localized in the canalicular or apical domain of the hepatocyte plasma membrane [30], and a loss of BSEP leads to intrahepatic cholestasis [34]. Mrp2, located in the canalicular membrane, can also induce bile acids to be secreted from the liver into bile [2]. NTCP, localized on the outside of the basolateral membrane, largely accounts for the uptake of conjugated bile acids (>80%) [11]. Meanwhile, Mrp3, localized at the basolateral membrane of hepatocytes and cholangiocytes, transports bile acids back to systemic circulation and excretion [2]. Our study showed that rhein influences the synthesis, metabolism, and transport of bile acids by affecting the expression of bile acid transporter genes. The multivariate statistical analysis demonstrated a notable distinction between the rhein group and the control; the main reason for this remarkable distinction was the variation in TCA and T-*α*-MCA levels.

Treatment with 1,000 mg/kg rhein notably repressed the expression of hepatic mRNA of FXR and SHP while remarkably elevating the expression of CYP7A1 mRNA, leading to increased bile acid synthesis. On the other hand, 1,000 mg/kg rhein also downregulated the expression of BSEP mRNA, resulting in decreasing secretion of bile acid from hepatocyte to bile duct, thus causing an increase in the levels of TBA in the liver (Figure 5(d), no statistical difference). While the high dose of rhein has the potential to increase the risk of cholestasis, thus, taking excessive doses of rhein over a long period should be avoided. Some studies have found that 120 mg/kg rhein can restore insulin secretion in mice [12]; 150 mg/kg rhein has good therapeutic effects on renal interstitial fibrosis in mice [35]. The high dose of rhein showed a good therapeutic effect, so this study increased the dose on the basis of 150 mg/kg of rhein and evaluated the safety of rhein under a high dose. However, the rhein in our study did not show obvious toxicity characteristics except that it increased liver TBA levels (due to the increase in T-*α*-MCA and TCA) and induced mild liver histological changes (inflammatory infiltration and fat droplets), suggesting that there were potential risks in taking high doses of rhein. T-*α*-MCA and TCA are worthy of attention when taking a high dose of rhein.

However, 1,000 mg/kg rhein is much higher than the clinical dose, and the chance of ingesting such a high dose of rhein is very low. Therefore, it is more meaningful to study the clinical dose of rhein. The 10 and 30 mg/kg rhein, close to clinical dose, significantly reduce serum bile acid levels, especially the UCBAs (Figure 6(a)), potentially resulting of the upregulation of the expression of liver FXR mRNA and MRP3 mRNA. Previous studies have reported that partial UCBAs such as CA, DCA, CDCA, and *β*-MCA displayed cytotoxicity due to high hydrophobicity [30]. t-CBAs (TCA, T-*α*-MCA) and g-CBAs, such as GCA, were less toxic [36]. Therefore, the decrease in the levels of serum UCBAs may be beneficial to the clinical use of medium and low dose of rhein. In comparison, rhein had more vigorous effect in elevation of FXR genes at dose of 30 mg/kg rather than that at dose of 10 mg/kg. However, these two doses of rhein had no significant effect on the regulation of SHP, CYP7A1, and CYP8B1 mRNA. It is suggested that medium and low doses of rhein mainly promote bile acid transport by upregulating Mrp3 mRNA expression, but has little effect on bile acid synthesis. It is also possible that FXR may affect bile acid synthesis by regulating the expression of FGF15 [32]. Rhein reduced bile acid levels at dose of 10 mg/kg and 30 mg/kg, while increased the amounts of bile acid at dose of 1000 mg/kg, probably because different doses of rhein had diverse effects on bile acid transporters. No significant abnormalities were found in either the 10 or 30 mg/kg groups. Therefore, rhein is relatively safe to use at a reasonable dosage and timing.

To conclude, the current data highlight rhein is relatively safe to use at a reasonable dosage and timing. Rhein, close to clinical dose, may promote bile acid transport and reduce bile acid accumulation by upregulating the expression of FXR mRNA and Mrp3 mRNA, potentially resulting in the decrease in serum UBCAs. However, the high dose of rhein inhibited the expression of FXR and BSEP mRNA, which may result in the accumulation of bile acids in liver.

## Figures and Tables

**Table 1:** Concentrations of bile acids in serum and liver after treated rats with rhein for 5 weeks.

	Control	Rhein		
	—	0.01 g/kg	0.03 g/kg	1 g/kg
Serum (ng/mL)				
T-*α*-MCA	383.6 ± 105.53	241.60 ± 111.27	146.40 ± 31.94^∗∗^	826.80 ± 256.84^∗^
TCA	18.62 ± 3.59	25.92 ± 12.84	13.16 ± 7.94	38.86 ± 16.74^∗^
THDCA	52.16 ± 17.66	46.36 ± 21.74	17.34 ± 3.13^∗^	9.59 ± 5.75^∗∗^
TDCA	121.44 ± 68.99	96.58 ± 47.50	113.88 ± 71.61	6.01 ± 3.51^∗^
GUDCA	2.66 ± 0.80	2.22 ± 1.13	1.32 ± 1.55	0.56 ± 0.34^∗∗^
GHDCA	14.74 ± 4.59	25.68 ± 13.82	14.13 ± 10.80	39.90 ± 20.29^∗^
GCDCA	27.5 ± 11.91	30.14 ± 21.74	7.15 ± 2.95^∗^	11.02 ± 3.37^∗^
GDCA	99.62 ± 26.50	24.70 ± 16.74^∗∗^	24.33 ± 10.72^∗∗^	16.35 ± 6.18^∗∗^
GCA	248.40 ± 78.62	594.60 ± 287.05	122.48 ± 33.96^∗^	294.26 ± 179.41
UDCA	387.4 ± 133.98	107.94 ± 54.82^∗∗^	95.08 ± 18.45^∗∗^	213.46 ± 112.03
DCA	723.2 ± 179.79	91.60 ± 36.62^∗∗^	127.00 ± 22.55^∗∗^	56.08 ± 34.09^∗∗∗^
*β*-MCA	1,385.4 ± 452.18	232.00 ± 26.94^∗∗^	270.20 ± 101.18^∗∗^	730.20 ± 197.87^∗^
CA	3,132 ± 1,231.91	281.80 ± 90.75^∗∗^	963.80 ± 365.11^∗^	3,740.00 ± 1,789.06
CDCA	1,517.4 ± 623.24	286.00 ± 162.30^∗∗^	269.68 ± 98.37^∗^	688.40 ± 312.90^∗^
Liver (*μ*g/g)				
T-*α*-MCA	8.11 ± 2.97	24.94 ± 6.33^∗∗^	29.49 ± 10.89^∗∗^	60.88 ± 23.79^∗∗^
TCA	48.95 ± 15.76	36.33 ± 15.75	38.80 ± 13.97	110.87 ± 32.40^∗∗^
THDCA	6.97 ± 3.09	4.27 ± 0.56	2.34 ± 1.23^∗^	2.46 ± 1.20^∗^
TDCA	25.86 ± 5.17	11.67 ± 3.24^∗∗^	9.93 ± 5.07^∗∗^	0.41 ± 0.25^∗∗∗^
GUDCA	0.80 ± 0.41	0.20 ± 0.06^∗^	0.12 ± 0.08^∗∗^	0.03 ± 0.03^∗∗^
GHDCA	1.53 ± 0.94	0.57 ± 0.32	0.14 ± 0.10^∗^	0.01 ± 0.00^∗^
GCDCA	2.78 ± 0.74	1.60 ± 0.61^∗^	0.66 ± 0.33^∗∗^	0.16 ± 0.10^∗∗^
GDCA	11.22 ± 2.41	2.29 ± 1.74^∗∗∗^	0.92 ± 0.74^∗∗∗^	0.02 ± 0.01^∗∗∗^
GCA	21.98 ± 14.85	13.79 ± 4.85	6.02 ± 3.28^∗∗^	4.64 ± 3.03^∗∗^
UDCA	0.23 ± 0.16	0.11 ± 0.04	0.15 ± 0.03	0.02 ± 0.01^∗^
DCA	1.11 ± 0.63	0.23 ± 0.30^∗^	0.63 ± 0.41	0.16 ± 0.25^∗^
*β*-MCA	2.07 ± 0.69	0.44 ± 0.15^∗^	0.57 ± 0.26^∗^	0.14 ± 0.08^∗^
CA	3.33 ± 1.00	0.22 ± 0.02^∗∗^	0.79 ± 0.55^∗∗^	0.35 ± 0.22^∗∗^
CDCA	0.26 ± 0.23	0.05 ± 0.01	0.10 ± 0.04	0.07 ± 0.02

Data are presented as the means ± SD concentrations in serum measured using UPLC-MS/MS on 5 rats. ^∗^*p* < 0.05, ^∗∗^*p* < 0.01, and ^∗∗∗^*p* < 0.001, compared with the control group of the same bile acid.

**Table 2:** Serum biochemical values of ALT, AST, ALP, *γ*-GT, TBIL, CHO, BUN, and CRE after rats treated with rhein for 5 weeks.

♂	Control	Rhein		
	—	0.01 g/kg	0.03 g/kg	1 g/kg
ALT (U/L)	36.20 ± 1.90	31.60 ± 3.10	34.20 ± 8.80	43.00 ± 7.40
AST (U/L)	94.40 ± 8.30	90.20 ± 19.20	91.60 ± 13.00	116.60 ± 26.70
ALP (U/L)	195.60 ± 24.50	202.80 ± 33.5	166.60 ± 12.5	186.40 ± 15.6
*γ*-GT (U/L)	0.54 ± 1.15	0.24 ± 0.13^∗^	0.32 ± 1.18^∗^	0.28 ± 0.21^∗^
TBIL (*μ*mol/L)	0.96 ± 0.18	0.72 ± 0.24	0.74 ± 0.32	0.56 ± 0.23^∗^
CHO (*μ*mol/L)	1.34 ± 0.18	1.26 ± 0.31	1.28 ± 0.35	1.21 ± 0.36
BUN (mmol/L)	6.67 ± 0.27	7.52 ± 1.51	6.35 ± 0.87	5.09 ± 0.6
CRE (*μ*mol/L)	26.6 ± 0.5	29.4 ± 2.3	25.4 ± 2.1	35.8 ± 12.7

Data are presented as means ± SD of 5 rats. ^∗^*p* < 0.05, compared with the control group.

## Data Availability

Most of the data used to support the findings of this study are included in the article; further data are available from the corresponding author upon request.

## Abbreviations

ALT: Alanine aminotransferase
ALP: Alkaline phosphatase
AST: Aspartate aminotransferase
BA: Bile acid
BSEP: Bile salt export pump
BUN: Blood urea nitrogen
CA: Cholic acid
CDCA: Chenodeoxycholic acid
CHO: Cholesterol
CRE: Creatinine
CYP7A1: Cholesterol 7*α*-hydroxylase
DCA: Deoxycholic acid
FXR: Farnesoid X receptor
GCA: Glycocholic acid
g-CBA: Glycin-conjugated bile acids
GCDCA: Glycochenodeoxycholic acid
GDCA: Glycodeoxycholic acid
GHDCA: Glycohyodeoxycholic acid
GUDCA: Glycoursodeoxycholic acid
HDCA: Hyodeoxycholic acid
Mrp2: Multidrug resistance-associated protein 2
Mrp3: Multidrug resistance-associated protein 3
MRM: Multiple-reaction monitoring
NTCP: Na^+^-dependent taurocholic cotransporting polypeptide
OPLS-DA: Orthogonal partial least-squares discrimination analysis
QC: Quality control
SHP: Small heterodimer partner
SPE: Solid-phase extraction
TBA: Total bile acid
TBIL: Total bilirubin
TCA: Taurocholic acid
t-CBAs: Taurine-conjugated bile acids
TDCA: Taurodeoxycholic acid
THDCA: Taurohyodeoxycholic acid
T-*α*-MCA: Tauro-*α*-muricholic acid
UCBAs: Unconjugated bile acids
UDCA: Ursodeoxycholic acid
UPLC: Ultraperformance liquid chromatography
VIP: Variable importance in projection
*β*-MCA: *β*-Muricholic acid
*γ*-GT: *γ*-Glutamyltransferase.

## Supplementary Materials

Supplementary Materials Table S1: the MRM acquisition parameters of bile acids and internal standards. The declustering potential (DP), collision energies (CE), collision cell exit potential (CXP), and entrance potential (EP) were showed. Table S2: linearity data and limits of detection (LOD) of bile acids in serum samples. Table S3: linearity data and limits of detection (LOD) of bile acids in liver samples. Table S4: precision, accuracy, and recovery data of bile acids in rat serum samples. Table S5: precision, accuracy, and recovery data of bile acids in rat liver samples. Table S6: PCR for the subsequent genes with the corresponding primers.
