# Supplementary material for: Effects of Rhein on Bile Acid Homeostasis in Rats
Source: Biomed Res Int. 2020 Nov 9;2020:8827955. doi: 10.1155/2020/8827955 (PMC7679202; doi:10.1155/2020/8827955)
Supplement: Supplementary Materials — Table S1: the MRM acquisition parameters of bile acids and internal standards. The declustering potential (DP), collision energies (CE), collision cell exit potential (CXP), and entrance potential (EP) were showed. Table S2: linearity data and limits of detection (LOD) of bile acids in serum samples. Table S3: linearity data and limits of detection (LOD) of bile acids in liver samples. Table S4: precision, accuracy, and recovery data of bile acids in rat serum samples. Table S5: precision, accuracy, and recovery data of bile acids in rat liver samples. Table S6: PCR for the subsequent genes with the corresponding primers. [file 8827955.f1.docx]

TABLE S1: The MRM acquisition parameters of bile acids and internal standards. The declustering potential (DP), collision energies (CE), collision cell exit potential (CXP) and entrance potential (EP) were showed.

| Compound | Parent ion  (m/z) | Daughter ion  (m/z) | DP  (volts) | EP (volts) | CE  (volts) | CXP  (volts) |
| --- | --- | --- | --- | --- | --- | --- |
| T-αMCA | 514.3 | 80 | -155 | -10 | -110 | -4 |
| TCA | 514.2 | 80 | -155 | -10 | -110 | -4 |
| UDCA | 391.3 | 391.3 | -105 | -10 | -30 | -9 |
| CDCA | 391.4 | 391.4 | -125 | -10 | -30 | -9 |
| DCA | 391.5 | 391.5 | -125 | -10 | -30 | -9 |
| β-MCA | 407.3 | 407.3 | -115 | -10 | -30 | -9 |
| CA | 407.2 | 407.2 | -115 | -10 | -30 | -9 |
| GUDCA | 448.3 | 74 | -115 | -10 | -70 | -4 |
| GHDCA | 448.2 | 74 | -125 | -10 | -65 | -4 |
| GCDCA | 448.4 | 74 | -125 | -10 | -65 | -4 |
| GDCA | 448.4 | 74 | -125 | -10 | -65 | -4 |
| GCA | 464.3 | 74 | -125 | -10 | -70 | -4 |
| THDCA | 498.4 | 80 | -145 | -10 | -110 | -4 |
| TDCA | 498.1 | 80 | -140 | -10 | -110 | -4 |
| HDCA | 401.2 | 401.2 | -115 | -10 | -30 | -9 |
| GDCA-d4 | 452.3 | 74 | -120 | -10 | -65 | -4 |
| DCA-d4 | 395.3 | 395.3 | -125 | -10 | -30 | -9 |
| TDCA-d4 | 502.3 | 80 | -175 | -10 | -110 | -4 |

TABLE S2: Linearity data and limits of detection (LOD) of bile acids in serum samples.

| Bile acid in serum | Calibration Range（ng/mL） | Correlation coefficient r^2^ | LOD（ng/mL） |
| --- | --- | --- | --- |
| T-αMCA | 10-5000 | 0.9925 | 0.49 |
| TCA | 10-500 | 0.9984 | 0.27 |
| UDCA | 10-5000 | 0.9900 | 0.39 |
| CDCA | 10-5000 | 0.9928 | 0.23 |
| DCA | 10-5000 | 0.9985 | 0.11 |
| β-MCA | 10-5000 | 0.9951 | 0.02 |
| CA | 10-5000 | 0.9909 | 0.20 |
| GUDCA | 10-500 | 0.9977 | 0.43 |
| GHDCA | 2-500 | 0.9933 | 0.36 |
| GCDCA | 10-500 | 0.9991 | 0.43 |
| GDCA | 10-500 | 0.9984 | 0.55 |
| GCA | 25-5000 | 0.9952 | 0.59 |
| THDCA | 10-500 | 0.9965 | 0.29 |
| TDCA | 10-500 | 0.9990 | 0.49 |

TABLE S3: Linearity data and limits of detection (LOD) of bile acids in liver samples.

| Bile acid in liver | Calibration Range（ng/mL） | Correlation coefficient r^2^ | LOD（ng/mL） |
| --- | --- | --- | --- |
| T-αMCA | 20-5000 | 0.9967 | 0.67 |
| TCA | 20-5000 | 0.9956 | 0.79 |
| UDCA | 20-10000 | 0.9980 | 0.64 |
| CDCA | 20-10000 | 0.9981 | 0.41 |
| DCA | 20-10000 | 0.9932 | 0.15 |
| β-MCA | 20-10000 | 0.9920 | 0.19 |
| CA | 20-10000 | 0.9922 | 0.23 |
| GUDCA | 20-10000 | 0.9948 | 0.35 |
| GHDCA | 20-10000 | 0.9933 | 0.51 |
| GCDCA | 20-10000 | 0.9932 | 0.72 |
| GDCA | 20-10000 | 0.9961 | 0.65 |
| GCA | 20-10000 | 0.9913 | 0.6 |
| THDCA | 20-5000 | 0.9967 | 0.29 |
| TDCA | 20-5000 | 0.9940 | 0.5 |
| HDCA | 20-10000 | 0.9962 | 1.44 |

TABLE S4: Precision, accuracy, and recovery data of bile acids in rat serum samples.

| Serum | Concentration (ng/ml) | Intra-day (n=6) | | Inter-day (n=6) | | Recovery (n=6, %) |
| --- | --- | --- | --- | --- | --- | --- |
|  |  | CV (%) | Accuracy (%) | CV (%) | Accuracy (%) |  |
| T-a-MCA | 10 | 2 | 97.6 | 3.1 | 100 | 100 |
|  | 100 | 6.3 | 112 | 5.1 | 108.8 | 108.8 |
|  | 1000 | 2.5 | 112 | 1.7 | 111 | 111 |
| TCA | 10 | 4.8 | 103.5 | 4.5 | 104 | 104 |
|  | 100 | 2.6 | 108 | 12.5 | 97.6 | 97.6 |
|  | 1000 | 2 | 106.5 | 2.2 | 108.3 | 108.3 |
| UDCA | 10 | 4.9 | 82.9 | 7.4 | 88.2 | 88.2 |
|  | 100 | 0.2 | 87 | 3.5 | 89.3 | 89.3 |
|  | 1000 | 1.7 | 93.6 | 2.7 | 95.7 | 95.7 |
| CDCA | 10 | 0.2 | 93.9 | 2.8 | 94.4 | 94.4 |
|  | 100 | 0.3 | 92.2 | 1.2 | 93.2 | 93.2 |
|  | 1000 | 0.7 | 101.5 | 2.5 | 103.8 | 103.8 |
| DCA | 10 | 2 | 90.4 | 6.1 | 95.2 | 95.2 |
|  | 100 | 2 | 105.5 | 1.4 | 105.8 | 105.8 |
|  | 1000 | 0.7 | 102.5 | 0.9 | 103.2 | 103.2 |
| β-MCA | 10 | 5.2 | 109 | 13.4 | 98 | 98 |
|  | 100 | 4.1 | 97.2 | 3 | 98.5 | 98.5 |
|  | 1000 | 0.7 | 105.5 | 0.5 | 105.8 | 105.8 |
| CA | 10 | 1.2 | 91.9 | 2 | 93 | 93 |
|  | 100 | 0.15 | 91.5 | 3.8 | 94.2 | 94.2 |
|  | 1000 | 2.9 | 92.7 | 3 | 94.8 | 94.8 |
| GUDCA | 10 | 2.4 | 90 | 4.2 | 93.2 | 93.2 |
|  | 100 | 0.4 | 93.9 | 5.9 | 98.2 | 98.2 |
|  | 1000 | 1.7 | 88.6 | 1.5 | 89.4 | 89.4 |
| GHDCA | 10 | 0.3 | 94.4 | 6.7 | 97.2 | 97.2 |
|  | 100 | 5 | 94.1 | 5.6 | 97.6 | 97.6 |
|  | 1000 | 2.2 | 86.2 | 1.3 | 86.2 | 86.2 |
| GCDCA | 10 | 0.5 | 96.1 | 4 | 99.3 | 99.3 |
|  | 100 | 1.1 | 99.2 | 3.7 | 101.6 | 101.6 |
|  | 1000 | 1.4 | 95.3 | 2.7 | 97.4 | 97.4 |
| GDCA | 10 | 2.3 | 85.3 | 1.4 | 85.5 | 85.5 |
|  | 100 | 0.7 | 102.5 | 3.4 | 105.3 | 105.3 |
|  | 1000 | 2 | 100.6 | 3.2 | 103.3 | 103.3 |
| GCA | 10 | 2 | 84.5 | 3.2 | 83.7 | 83.7 |
|  | 100 | 0.1 | 93.2 | 3.5 | 96 | 96 |
|  | 1000 | 1.3 | 106 | 0.7 | 106 | 106 |
| THDCA | 10 | 3.4 | 99.6 | 3.9 | 99.8 | 99.8 |
|  | 100 | 0.5 | 102 | 1.8 | 103.3 | 103.3 |
|  | 1000 | 0.3 | 98.7 | 2.5 | 100.8 | 100.8 |
| TDCA | 10 | 1.6 | 98.4 | 6.8 | 98.6 | 98.6 |
|  | 100 | 2 | 107.5 | 2 | 106.5 | 106.5 |
|  | 1000 | 0.6 | 104.5 | 1.4 | 105.8 | 105.8 |

TABLE S5: Precision, accuracy, and recovery data of bile acids in rat liver samples.

| Liver | Concentration (ng/ml) | Intra-day (n=6) | | Inter-day (n=6) | | Recovery (n=6, %) |
| --- | --- | --- | --- | --- | --- | --- |
|  |  | CV (%) | Accuracy (%) | CV (%) | Accuracy (%) |  |
| T-a-MCA | 100 | 4.4 | 103.1 | 7.3 | 114.6 | 109.2 |
|  | 1000 | 2.5 | 116.8 | 3.4 | 115 | 114.7 |
|  | 10000 | 0.5 | 90 | 3.6 | 90.5 | 90.6 |
| TCA | 100 | 3.2 | 114.5 | 4.2 | 117.8 | 116.8 |
|  | 1000 | 2.6 | 120 | 3.8 | 115.7 | 114 |
|  | 10000 | 2.6 | 85.6 | 3.9 | 86.2 | 86.6 |
| UDCA | 100 | 2.5 | 120.7 | 1.8 | 122.1 | 119.6 |
|  | 1000 | 1.8 | 109.2 | 2.1 | 107.2 | 107 |
|  | 10000 | 2.3 | 107.2 | 4.3 | 109.2 | 110 |
| CDCA | 100 | 1.5 | 121 | 2.3 | 125.5 | 109.1 |
|  | 1000 | 0.5 | 101 | 1.3 | 99.4 | 104.2 |
|  | 10000 | 1 | 107.2 | 2.6 | 60.8 | 98.9 |
| DCA | 100 | 1.4 | 114.2 | 2.3 | 116.5 | 117 |
|  | 1000 | 0.5 | 106.3 | 1.3 | 105.8 | 105.8 |
|  | 10000 | 1 | 83.8 | 2.6 | 85.7 | 86.1 |
| β-MCA | 100 | 4.4 | 132.5 | 4.6 | 131.9 | 99.9 |
|  | 1000 | 0.8 | 113.4 | 3.2 | 110.6 | 110 |
|  | 10000 | 1.8 | 62.6 | 2.9 | 64 | 64.2 |
| CA | 100 | 2.5 | 134 | 2.9 | 131.7 | 99.9 |
|  | 1000 | 1.1 | 115.2 | 2 | 113.9 | 113.7 |
|  | 10000 | 1.6 | 93.1 | 2.6 | 95 | 95.4 |
| GUDCA | 100 | 3.7 | 112.8 | 3.6 | 115.2 | 125.7 |
|  | 1000 | 2.9 | 105.8 | 3.5 | 106.5 | 106.3 |
|  | 10000 | 1.7 | 84.7 | 3.6 | 86.9 | 87.3 |
| GHDCA | 100 | 3.7 | 112.8 | 3.6 | 115.2 | 93.7 |
|  | 1000 | 2.9 | 105.8 | 3.5 | 106.5 | 82.2 |
|  | 10000 | 1.7 | 84.7 | 3.6 | 86.9 | 86.2 |
| GCDCA | 100 | 3.5 | 116.2 | 3.2 | 117.3 | 117.2 |
|  | 1000 | 3.2 | 107.5 | 2.8 | 106 | 105.4 |
|  | 10000 | 1.7 | 90.3 | 3.7 | 92.8 | 93.4 |
| GDCA | 100 | 2.7 | 112.6 | 3.5 | 112.8 | 112.6 |
|  | 1000 | 3.3 | 106.7 | 3.1 | 105.8 | 104.6 |
|  | 10000 | 1.8 | 91.7 | 3.8 | 93.8 | 94.3 |
| GCA | 100 | 4.7 | 117.6 | 5.1 | 121.1 | 113 |
|  | 1000 | 4.2 | 104.3 | 5.4 | 102.4 | 101.8 |
|  | 10000 | 2.2 | 66.3 | 4.8 | 69 | 69.7 |
| THDCA | 100 | 4.7 | 108.6 | 5.1 | 116.2 | 115.3 |
|  | 1000 | 5 | 120.6 | 5.2 | 117 | 115.3 |
|  | 10000 | 0.8 | 83.5 | 3.2 | 85.2 | 85.7 |
| TDCA | 100 | 3 | 110.4 | 5 | 114.5 | 116 |
|  | 1000 | 3.9 | 108.8 | 3.3 | 106.3 | 106.1 |
|  | 10000 | 2.6 | 80.6 | 3.7 | 82.3 | 82.9 |

TABLE S6: PCR for the subsequent genes with the corresponding primers.

| Gene | Primer |
| --- | --- |
| FXR | Forward: 5’-CAGCAGACCCTCCTGGATTA-3’ |
|  | Reverse: 5’-TCTTCGTGGTCCAGTGTCTG-3’ |
| CYP7A1 | Forward: 5’-TGCCTTCTGTTACCGAGTGATGTT-3’ |
|  | Reverse: 5’-ACCGGCAGGTCATTCAGTTGCACT-3’ |
| BSEP | Forward: 5’-TCTATGGACTCTGCTTTGCCTTTT-3’ |
|  | Reverse: 5’-GCCCAGACCTTCGTAGGCTA-3’ |
| NTCP | Forward: 5’-CCCTGATGCCCTTCTCTGG-3’ |
|  | Reverse: 5’-GAATCCTGTTTCCATGCTGATG-3’ |
| Mrp2 | Forward: 5’-TGGAGTTGGCTCACCTCAGATC-3’ |
|  | Reverse: 5’-CTAGAGCTCTGTGTGATTCACATTTTCA-3’ |
| Mrp3 | Forward: 5’-GTGCTGAAGAATTTGACTCTG-3’ |
|  | Reverse: 5’-GACCAGGACCCGGTTGTAGTC-3’ |
| GAPDH | Forward: 5’-CCTGGAGAAACCTGCCAAGTAT-3’ |
|  | Reverse: 5’-AGCCCAGGATGCCC TTTAGT-3’ |
